# Supplementary material for: Cognitive and behavioral effects of whole brain conventional or high dose rate (FLASH) proton irradiation in a neonatal Sprague Dawley rat model
Source: PLoS One. 2022 Sep 16;17(9):e0274007. doi: 10.1371/journal.pone.0274007 (PMC9481014; doi:10.1371/journal.pone.0274007)
Supplement: Attachment — Submitted filename: PONE-D-22-04666 R1 response to reviewers.docx [file pone.0274007.s002.docx]

Reviewer 1: Provide additional explanation for the limited protective effect of FLASH, which incorporates findings from other groups. The results of the ELISA and Western blot analysis should also be mentioned in the discussion.

**Reply. A new paragraph has been added to the Discussion on the protective effects. We argue that the 5-FLASH group produced sparing when there were deficits in the 5-Conv group. We have also incorporated the results of the western and ELISAs.**

- Reviewer 2: Please provide a rationale for the order of the behavioural tasks particularly in regards to the ASR and TSR being close to the start of the testing battery

**Reply:** **The following has been added: “A behavioral battery of tests was used to reduce the number of rats used, provide a better characterization of effects for this initial experiment (41), and to be more translatable to human studies that also employ test batteries of different cognitive domains. While test order may have some effect on subsequent behaviors (42), having a day between different tests alleviated many of these issues (43). We also minimized these effects by performing tasks from the least stressful to most stressful (with the exception of the CWM mirror version). While fear potentiated startle can cause changes in dopamine (44), the startle response itself is not known to produce long-term dopamine changes (45).”**

- Reviewer 3:

1) While the days on which the behavioral tests are described in each section, it would be helpful to have a timeline figure that shows the organization of these days over time. This gives the reader a better idea of the differences in time needed for each behavioral test.

**Reply: A timeline is now provided.**

2) The authors should comment on the potential for numerous behavioral tests to interact with or cause changes in future behavioral performance. For example, they noted that the behavioral tests were run from assumed least stressful to most stressful, but it is possible that learning one test could alter learning of subsequent behavioral tests. Given that only one test was repeated and the results were different from the first assessment (5 Gy FLASH), would differences in performances of the other groups potentially be reversed if the rats were re-tested?

**Reply: There were 2 different versions of the Cincinnati water maze (CWM) used in this experiment. For the first version, the 5-FLASH group was delayed in learning, as demonstrated by the interaction of exposure x day, but performed equally to the controls by the end of the test. We now make this point better in the Discussion. The mirror version of the CWM while similar requires a completely new set of turns and the 5-FLASH group performed similarly to controls. We have now made this clear that there is consistency in the effects that were observed for the 5-FLASH group.**

3) There were limited effects on the neuronal markers chosen (DRD1, DRD2, DAT, TH, NMDA-NR1). The authors should comment on the lack of measurements typical of other FLASH studies, such as neuroinflammation and neurogenesis and what role these processes might play in the differences between these groups.

**Reply: We have added the following: A number of studies examining the effect of FLASH in the brain of adult mice have focused on markers of overt neurotoxicity such as glial fibrillary acidic protein staining or bromodeoxyuridine uptake (16-18). However, in this study the approach was to examine the neurotransmitter system related to the functional changes observed in these rats. Therefore the focus was on the dopamine system because of the changes in the CWM and the NMDA receptor subunit 1 (NMDA-NR1) in the hippocampus to confirm the MWM results.**

When submitting your revision, we need you to address these additional requirements.

1. Please ensure that your manuscript meets PLOS ONE's style requirements, including those for file naming. The PLOS ONE style templates can be found at

**Reply: style changed as required.**

2. Thank you for stating the following in the Funding Section of your manuscript:

“This research was supported by a Research Agreement from Varian, a Siemens Healthineers company. Additional funding support included research support from the TQL Foundation, Cincinnati, OH and Fund the Cure Next Door Foundation.”

We note that you have provided funding information that is not currently declared in your Funding Statement. However, funding information should not appear in the Acknowledgments section or other areas of your manuscript. We will only publish funding information present in the Funding Statement section of the online submission form.

**Reply: Funding-related text was removed from the manuscript. The Funding Statement should reads as follows:**

**This research was supported by a Research Agreement from Varian, a Siemens Healthineers company. Additional funding support included research support from the TQL Foundation, Cincinnati, OH and Fund the Cure Next Door Foundation.**

Please include your amended statements within your cover letter; we will change the online submission form on your behalf.

3. Thank you for stating the following financial disclosure:

“This research was supported by a Research Agreement from Varian, a Siemens Healthineers company. Additional funding support included research support from the TQL Foundation, Cincinnati, OH and Fund the Cure Next Door Foundation.”

Please state what role the funders took in the study. If the funders had no role, please state: ""The funders had no role in study design, data collection and analysis, decision to publish, or preparation of the manuscript.""

If this statement is not correct you must amend it as needed.

Please include this amended Role of Funder statement in your cover letter; we will change the online submission form on your behalf.

**Reply: The financial disclosure should read as follows:**

**This research was supported by a Research Agreement from Varian, a Siemens Healthineers company. Additional funding support included research support from the TQL Foundation, Cincinnati, OH and Fund the Cure Next Door Foundation. The funders had no role in study design, data collection and analysis, decision to publish, or preparation of the manuscript. Feedback was provided by Varian for the initial manuscript submission.**

4. Thank you for stating the following in the Competing Interests section:

“These experiments were funded by Varian, a Siemens Healthineers company that granted the authors intellectual freedom to publish the data.”

Please confirm that this does not alter your adherence to all PLOS ONE policies on sharing data and materials, by including the following statement: ""This does not alter our adherence to PLOS ONE policies on sharing data and materials.” (as detailed online in our guide for authors http://journals.plos.org/plosone/s/competing-interests). If there are restrictions on sharing of data and/or materials, please state these. Please note that we cannot proceed with consideration of your article until this information has been declared.

**Reply: The Competing Interests statement should read as follows:**

**These experiments were funded by Varian, a Siemens Healthineers company that granted the authors intellectual freedom to publish the data. This does not alter our adherence to PLOS ONE policies on sharing data and materials.**

Please include your updated Competing Interests statement in your cover letter; we will change the online submission form on your behalf.

5. Please include captions for your Supporting Information files at the end of your manuscript, and update any in-text citations to match accordingly. Please see our Supporting Information guidelines for more information: http://journals.plos.org/plosone/s/supporting-information.

**Reply: There are no in text citations to supporting information.**
